# Monitoring of tumor growth and vascularization with repetitive ultrasonography in the chicken chorioallantoic-membrane-assay

**DOI:** 10.1038/s41598-020-75660-y

**Published:** 2020-10-29

**Authors:** Jonas Eckrich, Philipp Kugler, Christoph Raphael Buhr, Benjamin Philipp Ernst, Simone Mendler, Jan Baumgart, Juergen Brieger, Nadine Wiesmann

**Affiliations:** 1 Department of Otorhinolaryngology, University Medical Center Mainz, Langenbeckstrasse 1, 55131 Mainz, Germany; 2 Department of Oral and Maxillofacial Surgery - Plastic Surgery, University Medical Center Mainz, Langenbeckstrasse 1, 55131 Mainz, Germany; 3 Translational Animal Research Center, University Medical Center Mainz, Hanns-Dieter-Hüsch-Weg 19, 55128 Mainz, Germany

**Keywords:** Cancer models, Ultrasound, Immunohistochemistry

## Abstract

The chorioallantoic-membrane (CAM)-assay is an established model for in vivo tumor research. Contrary to rodent-xenograft-models, the CAM-assay does not require breeding of immunodeficient strains due to native immunodeficiency. This allows xenografts to grow on the non-innervated CAM without pain or impairment for the embryo. Considering multidirectional tumor growth, limited monitoring capability of tumor size is the main methodological limitation of the CAM-assay for tumor research. Enclosure of the tumor by the radiopaque eggshell and the small structural size only allows monitoring from above and challenges established imaging techniques. We report the eligibility of ultrasonography for repetitive visualization of tumor growth and vascularization in the CAM-assay. After tumor ingrowth, ultrasonography was repetitively performed *in ovo* using a commercial ultrasonographic scanner. Finally, the tumor was excised and histologically analyzed. Tumor growth and angiogenesis were successfully monitored and findings in ultrasonographic imaging significantly correlated with results obtained in histological analysis. Ultrasonography is cost efficient and widely available. Tumor imaging *in ovo* enables the longitudinal monitoring of tumoral development, yet allowing high quantitative output due to the CAM-assays simple and cheap methodology. Thus, this methodological novelty improves reproducibility in the field of in vivo tumor experimentation emphasizing the CAM-assay as an alternative to rodent-xenograft-models.

## Introduction

Chicken eggs have been used as an experimental tool since the late nineteenth century^1^. Further development of the method led to the identification of the chorioallantoic-membrane (CAM) as an easily accessible, well-vascularized anatomical structure suitable for versatile experimentation. The CAM is an extraembryonic membrane that is formed by the partial fusing of the chick’s chorion and its allantois during the embryonal development. As previously described in detail by D. Ribatti^2^, the allantoic vesicle enlarges very rapidly in the timeframe from day four till ten of incubation. During this process, the mesodermal layer of the allantois fuses with the adjacent mesodermal layer of the chorion. Hence, the CAM consists of the chorionic epithelium, a highly vascularized mesodermal layer and the allantoic epithelium. The CAM´s vascular network is connected to the embryonic bloodstream by two allantoic arteries as well as one allantoic vein. During the process of embryonal development, the CAM´s surface as well as the vascular network rapidly expand ^2–4^. Growth of the CAM as well as it’s differentiation starts to stagnate on day 11 further leading to a fully developed and differentiated CAM on day 13^2, 4^.

Regarding its functions, the CAM can be considered as an equivalent to the mammalian placenta. Besides enabling the exchange of respiratory gases, it also draws calcium from the eggshell for the embryonal bone development and regulates the acid–base homeostasis of the embryo as well as the reabsorption of ion and H_2_O from the allantoic fluid^5^.

Motivated by ethical concerns regarding the Draize rabbit eye test, Luepke identified the CAM as an alternative testing method in toxicological research^6^. The CAM is accessed for experimentation by partial removal of the eggshell. Exposition of the CAM for research allowed further establishment of this model as a versatile research tool for the analysis of angiogenesis^7^, biomaterial research^8^, toxicology^9–15^, wound healing^16^, bone regeneration^12^ and tumor development^16^.

For experimental tumor research, the CAM as a highly vascularized, non-innervated, extra-embryonic membrane has proven to be well-suited for inoculation of tumor cells. The high density of blood vessels creates an ideal milieu for tumor growth due to the ubiquitous supply of oxygen, nutrients, and growth factors^4^. Since the early twentieth century different authors have had success with the cultivation of solid tumors in the chicken egg and onto the CAM^17^. Ingrowth of blood vessels into the tumor has been described to start from day two to five after inoculation^18–21^.

Due to the natural absence of specific immune system in the hen’s egg till day 14 of development, the implantation of xenografts onto the CAM does not require artificial induction of immunodeficiency^22^.

In contrast, the sufficient immune system in rodent models leads to the rejection of xenografts. Consequently, rodent strains with severe immunodeficiency or a humanized immune system are a prerequisite for xenograft transplantation^23^. Common rodent- xenograft-models comprise mice and rats with absence of B-cells (nude mice/rats) or severe combined immunodeficiency (SCID)^24–26^.

Apart from ethical issues regarding the health of host animals, the use of rodent-xenograft-models requires specialized, very effortful and costly conditions suitable for keeping and breeding of host animals. In contrast, the CAM-assay is a comparably simple methodology with moderate space requirements regarding incubation. This allows a high quantitative output and a good reproducibility without the need for excessive costs, personnel, or equipment^27, 28^.

In rodent models, the implantation of the xenograft, monitoring of tumor growth, and -vascularization as well as any therapeutical interventions result in either stress, physical discomfort, pain or ultimately death for the host animal. In opposition to these constraints, the CAM has no nociceptive innervation. Moreover, the whole chick embryo is unable to experience pain until day 14 of incubation due to the incomplete development of the nervous system^2^.

In countries with high standards for animal care and research, using the CAM assay faces very low bureaucratic hurdles compared to in vivo rodent experiments since experimentation with the CAM assay does neither require approval by a governmental organization nor an ethics committee for animal experimentation as long as the chickens are not hatched^2, 29^.

Multiple tumor entities have shown to grow sufficiently on the CAM^20, 30–32^. Observable parameters in the field of tumor research with this model are horizontal tumor size and vascularization of the growing tumor^33^. Without any tissue layer between the observer and the implanted tumor, the malignant cells are fully accessible for observation and manipulation. Tumor angiogenesis and vasculogenesis can further be visualized using in vivo microscopy^16^ in the CAM adjacent to the tumor.

Monitoring of three-dimensional tumor size and growth however, might be the main methodological limitation of the CAM-assay in the field of tumor experimentation. Although a direct view on the CAM is possible after partial shell removal, the microscopical evaluation of tumor size and tumor vascularization is limited by the opacity of the surrounding eggshell as well as the autofluorescence of the solid tumor in fluorescence microscopy. In our experience, the tumor often grows deep into the CAM, resulting in a massive increase in tumor volume without major changes in lateral diameter. Furthermore, the movements of the chicken embryo further impede sufficient and reproducible microscopical investigation *in ovo*.

An end-point analysis can be realized by caliper measurements after excision or tumor fixation and preparation of histological slides. However, the high variation in directional growth as well as artefacts acquired during the fixation process limit the interpretability of these findings. Furthermore, an end-point analysis has obvious limitations for the interpretation of therapeutical effects in tumor experimentation, especially when the a.m. variability of tumor growth is taken into account.

Imaging techniques like micro-computed tomography (CT) and micro-magnetic resonance imaging (MRI) might be suitable for three-dimensional visualization of the tumor. However, time expenditure, high costs, availability of equipment as well as the resolution capacity needed for the visualization of the small structural size of the egg^34^ are obvious methodological limitations. The high density of the eggshell causes radio-opacity requiring the application of contrast agents for sufficient visualization of structures within the eggshell^35^. These limitations can be overcome by intravascular application of markers and contrast agents. Yet, intavascular application of contrast agents is a challenging task due to the small diameter of the vessels on the CAM. Furthermore, intravascular application often results in excessive bleeding, leading to an increased dropout rate, impaired comparability, a deteriorated experimental conditions and a potential inter-operator bias.

Thus, up until now, the absence of an accessible and sufficient three-dimensional imaging technique, suitable for adequate visualization of tumor development limited the applicability of the CAM-assay for various investigations in the field of tumor research.

Ultrasonography utilizes a piezoelectric crystal, which transmits and receives soundwaves usually in a range between 1 and 40 MHz. The live image is calculated by comparing the transmitted sound waves to the recurrent ones. Obvious key advantages are its widespread availability and its simple test setup. In addition, time and financial expenditure are far below equivalent imaging techniques like CT and MRI. Furthermore, ultrasonography does not influence or affect the experimental animal. Due to the absence of pain or suffering, no anesthesia is needed. In experimental research, ultrasonography has already been used successfully for the quantification of tumor size in different in vivo models like mice^36^. Furthermore, *in ovo* ultrasonography has been utilized to investigate chicken embryology and especially the chick’s heart development^34, 37^. Taking these findings into consideration and searching for a suitable imaging method to quantify the size of tumors grown on the CAM in three dimensions, we tested the eligibility of repetitive color-duplex-ultrasonography for the analysis of tumor growth and tumor vascularization.

According to the Russell’s and Burch’s “Principles of Humane Experimental Technique” the reasonable use of the CAM-assay contributes to the refinement of animal experiments by minimizing pain and suffering of animals. As this methodology diversifies the applicability of the CAM–assay as a replacement for homologue rodent experiments, this model therefore meets the ethical obligations to reduce, refine, and replace (3Rs) animal usage in tumor research.

Specifically, this study evaluates the suitability of both single time as well as repetitive ultrasonography of tumors grown on the CAM for the quantification of tumor size, tumor growth and the evaluation of tumor vascularization. Comparative dropout rates were determined. Moreover, results obtained in the ultrasonographic measurements were compared to the corresponding histological specimen using immunohistochemical analysis for the determination of tumor size and tumor vascularization. Finally, an exemplary comparative mathematical analysis of the costs of the *in ovo* tumor model and the in vivo tumor model was conducted.

## Methods

### Eggs and tumor development

White Leghorn hens’ eggs were placed horizontally in an incubator (Brutmaschinen-Janeschitz GmbH, Hammelburg, Deutschland) at 37.5 °C. To allow exposure of the CAM by detaching the membrane from the eggshell, on day 3 of incubation, 6 ml albumen was removed by aspiration with a sterilized syringe. The shell was subsequently opened with sterilized scissors, parts of the shell were removed and the CAM was exposed. After opening the eggshell, the aperture was covered with PARAFILM® (Bemis Company Inc., Neenah, Wisconsin, USA) to avoid evaporation. Cultivation of the liver cancer HuH7 tumor cells^38^ on the CAM started by day 7.

One day before placement onto the CAM the HuH7 tumor cells were harvested by tryptic digestion from the cell culture flask, counted with a Neubauer counting chamber, and distributed in 1.5 ml tubes (5 Mio. cells per egg). After centrifugation at 1400 rpm for 10 min the supernatant was removed, and the cell pellet was subsequently suspended in 20 µl of ice cooled Matrigel™ (Corning™, Brumath, France) similar to procedures previously published^20, 27^. The five million cells were then incubated for 30 min on a 6-well plate at 37.5 C (Greiner, bio-one International GmbH, Kremsmünster, Austria) until the Matrigel™ had hardened to a firm consistency. The 3D cell culture was then covered with culture medium, and finally incubated overnight.

Upon placement on the CAM a well vascularized spot was selected and the CAM was carefully incised using a single-use-scalpel (Feather, Dr. Junghans Medical GmbH, Bad Lausick, Germany) over a distance of approximately 0.5 cm opening the upper cellular layer. This incision has shown to have a positive effect on tumor inoculation as well as vessel ingrowth into the tumor without having relevant effects on the dropout rate. The 3D culture was subsequently placed onto the incision and 20 µl of Matrigel™ was pipetted onto the culture for protection against cell desiccation and to further immobilize the tumor on the CAM. The eggs were then incubated as mentioned above.

### Ultrasonography

**Figure 1:**
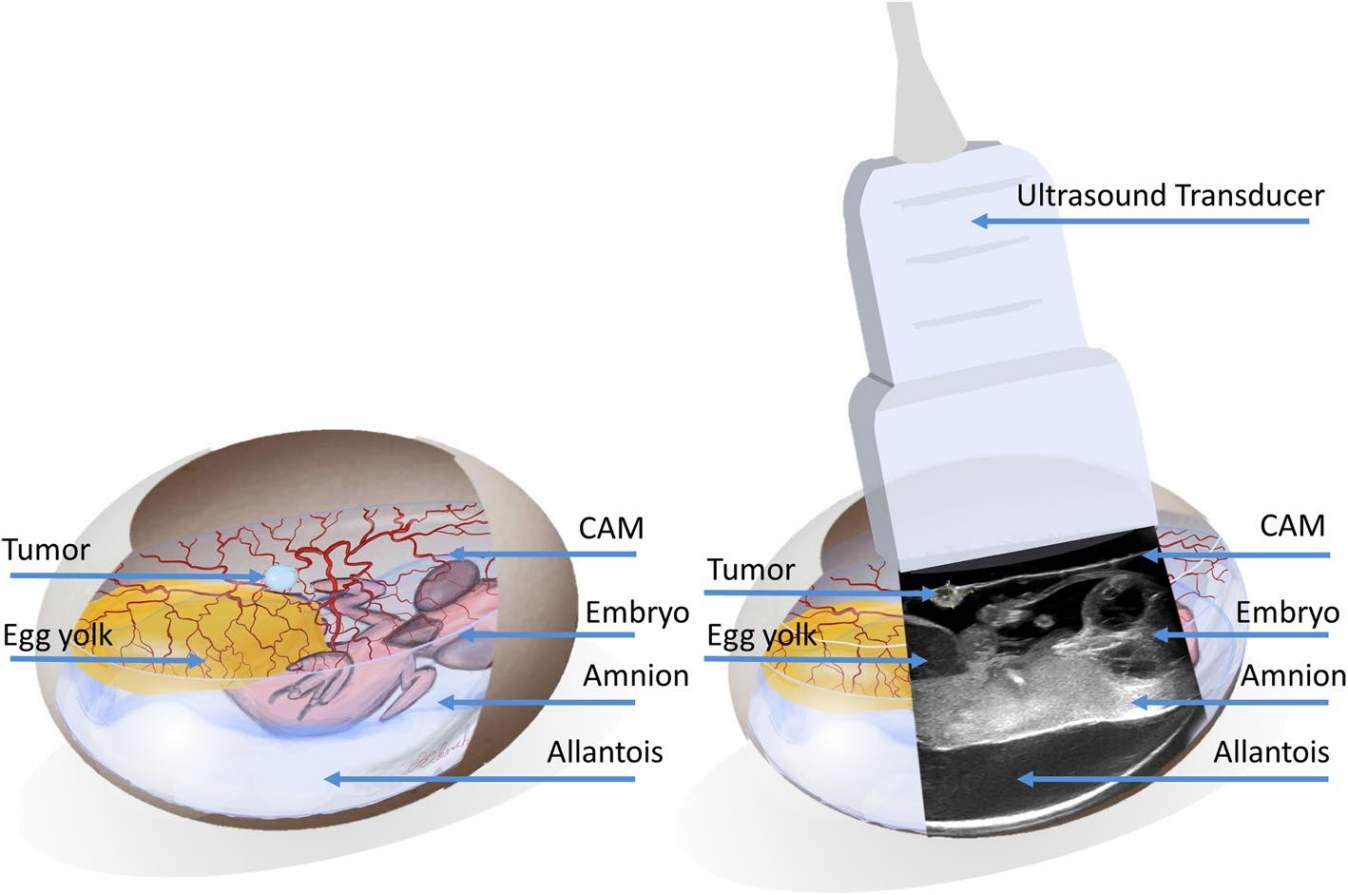
Visualization of the tumor and adjacent anatomical structures *in ovo:* Visualization of the tumor and adjacent anatomical structures in ovo. Further to the ultrasonographic overview in resemblance, tumors where magnified and focused upon to allow maximum precision regarding size and vascularization measurements.

Ultrasonographic evaluations were performed by highly experienced sonographic examiners (i.e. DEGUM Level III) to ensure the best possible investigational quality. Starting from day 12 of incubation (5 days after tumor inoculation) the GE Healthcare Ultrasound LOGIQ E9 (GE Healthcare Little Chalfont, UK) 15 MHz linear transducer was used in the B-Mode (Gain 35) for ultrasonographic imaging (Fig. 1). Instead of ultrasound gel the space between the CAM and shell opening was filled with an average of 4 ml NaCl 0.9% to allow transduction of ultrasound waves. Tumors were then visualized in both longitudinal and transversal axes to enable a three-dimensional quantification of the tumor size. The respective image was frozen using the “Freeze” function and the tumor length, width and thickness was measured and documented.

Color-duplex-sonography was carried out using the same methodology while the built-in Duplex mode enabled visualization of the vessels within the tumor. Video sequences were saved for offline analysis.

For repetitive measurements, the same procedure was carried out on days 12, 13, and 14 respectively. The NaCl 0.9% solution was removed after each measurement using an electrical pipette (INTEGRA Biosciences GmbH, Biebertal, Germany) with a 10 ml sterile tube (Greiner CELLSTAR® serological pipette, Greiner AG, Bischofsheim, Germany).

### Immunohistochemistry

After completion of the study protocol the embryo was sacrificed by decapitation. The CAM bearing the tumor was then excised with sterilized surgical scissors and placed onto filter paper stripes. The longitudinal and transversal axis were marked on the paper, the tumor-bearing CAM was transferred into a plastic cassette (Carl Roth GmbH + Co. KG, Karlsruhe, Germany), immobilized and put into a formalin solution (4%) (VWR International bvba, Leuven, Belgium) for 24 h. Afterwards, the plastic cassette was removed from the 4% formalin solution, washed three times with purified water for 20 min each, and incubated in isopropanol solution with increasing concentrations (80%/90%/100%) for 1 h each. The cassette was then washed with purified water and incubated in xylene (AppliChem GmbH, Darmstadt, Germany) for 24 h. Each specimen was imbedded in paraffin and cut into 5 µm slides with a microtome (Leica CM1900, Leica Biosystems Nussloch GmbH, Nussloch, Germany) according to the marking previously placed on the slides.

For HE stains, Paraffin was removed from the slide and the specimen was incubated in Mayer’s Hemalum Roth (Carl Roth GmbH + Co. KG, Karlsruhe, Germany) for 5 min. Subsequently, each slide was again washed in purified water and incubated in eosin (Merck KGaA, Darmstadt, Germany) for 2 min. Slides were then incubated in isopropanol solution with increasing concentrations (80%/90%/100%) for 2 min each and xylene for 10 min. Finally, slides were prepared for microscopy by embedding the specimen in Eukitt® (Sigma-Aldrich, St. Louis, Missouri, USA).

Immunohistochemical staining for Alpha Smooth Muscle Actin (Alpha-SMA) allowed visualization of vessels within the CAM. Slides were dewaxed with xylene and isopropanol solutions with decreasing concentrations (100%/90%/80%/70%) for 5 min each and cooked in citrate buffer (pH 6.0) for 30 min. After preparation slides were incubated with monoclonal Alpha-SMA antibodies (A2547, Sigma-Aldrich, St. Louis, Missouri, USA) dissolved in (1/1500) phosphate-buffered-saline (PBS) + 1% bovine serum albumin (BSA). As secondary antibody biotinylated polyclonal goat anti-mouse immunoglobulin (P 0447, Dako Denmark A/S, Glostrup, Denmark) was added and visualized using horseradish peroxidase-conjugated streptavidin (1/250) (Dako Denmark A/S, Glostrup, Denmark). Finally, slides were again prepared for microscopy by embedding the specimen in Eukitt ® (Sigma-Aldrich, St. Louis, Missouri, USA).

### Microscopical analysis

Microscopical slides were investigated using the Nikon Eclipse TE2000 Inverted Microscope (Nikon Corp. Chiyoda, Japan) and digitalized by transferring the image from the inbuilt camera system (Nikon's DS-Fi3, Nikon Corp. Chiyoda, Japan) into Nikon’s analysis software NIS-Elements (Nikon Corp. Chiyoda, Japan).

### Data management and off-line analysis

Tumor diameters, measured in ultrasonographic images were transferred into EXCEL sheets (Microsoft Corp., Redmond, WA, USA). For determination of tumor vascularization, the video files of color-duplex-ultrasonography were exported and the presence and intensity of intratumoral vessels on the CAM was rated in a three step rating system (0 = no intratumoral vascularization, 1 = moderate intratumoral vascularization, 2 = intense intratumoral vascularization) by two blinded otorhinolaryngological specialists (J.E., B.E.), experienced in clinical ultrasonographic diagnostics independently. Ratings were then evaluated for concordance and reevaluated in case of discrepancy until a clinical consensus was reached between both investigators. Results were again inserted into EXCEL sheets.

The images of the HE-stained tumors were used to determine tumor size by measuring the diameters in both sagittal and transversal planes by laboratory personnel blinded to the results of the ultrasonographic imaging. To evaluate tumor vascularization Alpha-SMA staining was used. Similarly, to ultrasonographic imaging, the amount of intratumoral vascularization was quantified in a three-step rating system (0 = no intratumoral vascularization, 1 = moderate intratumoral vascularization, 2 = intense intratumoral vascularization).

As tumors grow in a rounded shape, calculation of estimated tumor volumes from the three diameters obtained in ultrasonography was realized using the triaxial ellipsoid formula (V = 4/3 × Pi x (0.5 × d1(sagittal)) x (0.5 × d2(transversal) × 0.5 x d3(coronar))).

For the longitudinally cut histological slides the approximated two-dimensional tumor area could be calculated by using the ellipsis formula (A = Pi x d1[sagittal] x d2[transversal]).

To further allow comparability of size quantification by ultrasonography with the size determined by histological analysis, the tumor area in ultrasonography was determined by insertion of the longitudinal and sagittal diameters in the above-mentioned ellipsis formula.

Success rates of tumor development were determined by calculating the percentage of solid tumors on the CAM on day 11 of incubation in relation to the number of eggs incubated on day 0. Accordingly, success rates regarding the possibility of ultrasonographic imaging in all three dimensions were calculated by dividing the eggs in which application of ultrasonography was possible by the number of CAMs with solid tumors.

### Statistical analysis

Column statistics as well as comparative statistical analysis were carried out using GraphPad Prism™ (GraphPad Software, Inc., La Jolla, CA, USA). Besides column statistics (mean, median, range, standard deviation, standard error, Gaussian distribution and confidence intervals 95) comparative analysis was carried out as follows: As most data sets regarding tumor volume and vascularization did not show a Gaussian distribution, correlation between tumor sizes determined in ultrasonography as well as histology was determined using the Spearman-correlation.

For longitudinal analysis of tumor growth as well as changes in vascularization determined in ultrasonography the Friedman repeated measures test was used to determine whether volumes significantly differed between the days of observation.

Differences in survival after repetitive ultrasonography were determined using the log-rank test as well as the Gehan-Breslow-Wilcoxon test.

### Cost analysis

For comparing the costs of the CAM assay to rodent tumor models, the average costs for mice represented by the Crt:NU(NCr)-Foxn1nu/nu line (male, 6 weeks old) for a duration of 14 days were calculated. As source data the median prices for chicken eggs of the main distributer in the specific region (Bio-Aufzucht LSL Rhein-Main GmbH, Dieburg, Germany) as well as mice according to the 3 available distributers for nude mice in the specific region (Janvier Labs, Paris, France; Charles River Wiga GmbH, Sulzfeld, Germany; Envigo RMS GmbH, Roßdorf, Germany) were taken into account. Costs for transport as well as the gross running costs, provided by Translational Animal Research Center, University Medical Center of the Johannes Gutenberg-University Mainz, Mainz, Germany were considered. The cost of keeping animals in our facility is on average the price of equivalent German facilities known to us. An amount of 75 eggs as well as 25 mice was taken as the average sample size.

## Results

### CAM-assay and tumor development

**Figure 2:**
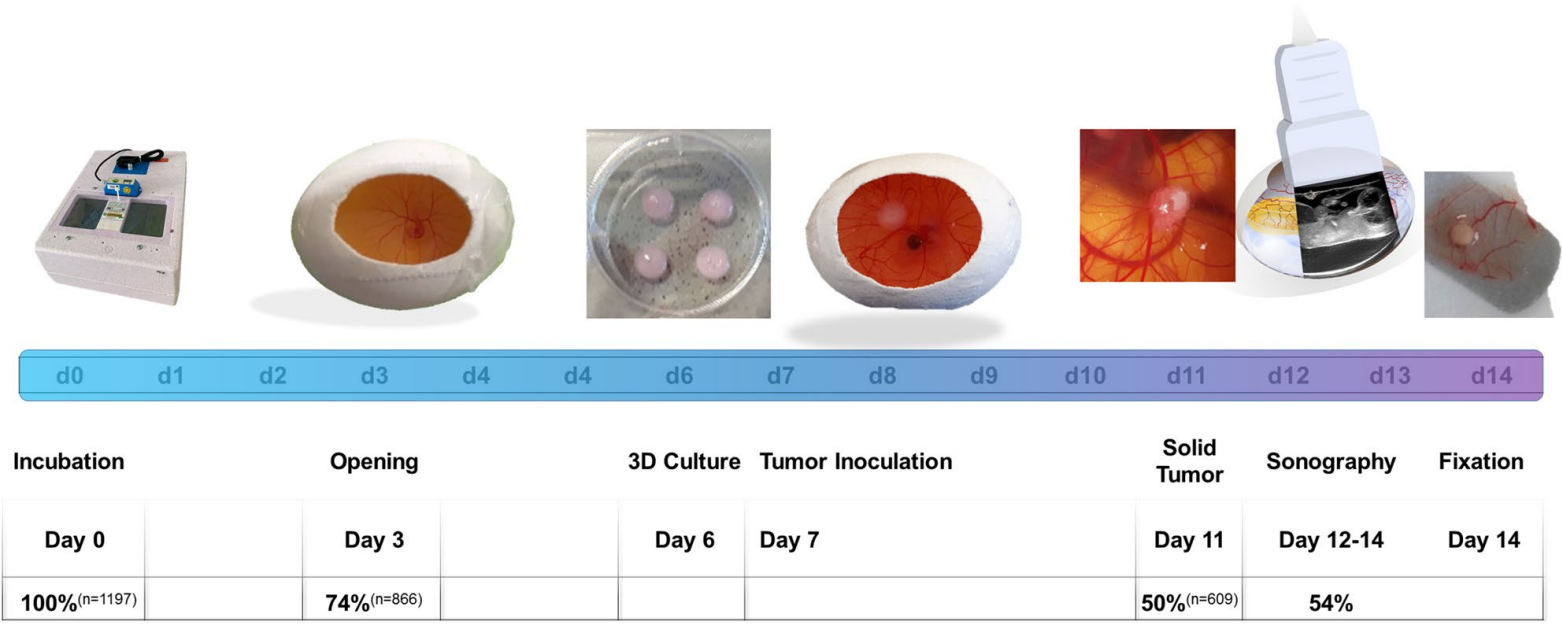
Timeline visualizing chronological steps of experimentation: The colored timeline shows the day of hatching after incubation (d0–d14). Breeding of the fertilized eggs started at day 0. Eggs were opened at day 3 and in vitro cultured tumors transferred on the CAM at day 7. Solid tumors were than analyzed from days 12 to 14 using ultrasonography. Of the 1197 eggs evaluated, 74% (n = 866) were fertilized and showed viability day 3 50% (609 eggs) successfully inoculated a tumor at day 11 not dropping out due to luxation, insufficient ingrowth or death of the embryo. Of the 186 eggs with solid tumors evaluated for ultrasonographic imaging, 100 eggs (54%) were suitable for sufficient ultrasonographic visualization.

As shown in Fig. 2 due to an extensive experience with this model we were able to retrospectively analyze a very high number of eggs (n = 1197) used for different studies in our lab. Of these eggs, upon opening of the eggshell 73.91% (± 8.90%) (n = 866) were fertilized and vital. After opening of the eggshell of the 866 eggs opened, 67.96% (± 10.68%) (n = 609) survived and were colonized with a tumor on day 7. Regarding the success rate of inoculation of (HUH7) tumors on the CAM, without any intervention till day 11, 50.39% (± 11.46%) of these 1197 eggs showed viability as well as sufficient ingrowth of the transplanted tumor localized within the observational window and suited for further investigation.

For this specific study regarding ultrasonographic analysis 189 eggs were randomly selected. Repetitive ultrasonography was performed on 36 eggs. 54.00% (± 29.47%) of the randomly selected eggs had a tumor positioning on the CAM eligible for sufficient ultrasonographic imaging and measuring of all three tumor diameters respectively. On 30 eggs, color-duplex- ultrasonography was performed additionally.

### Tumor size

**Figure 3:**
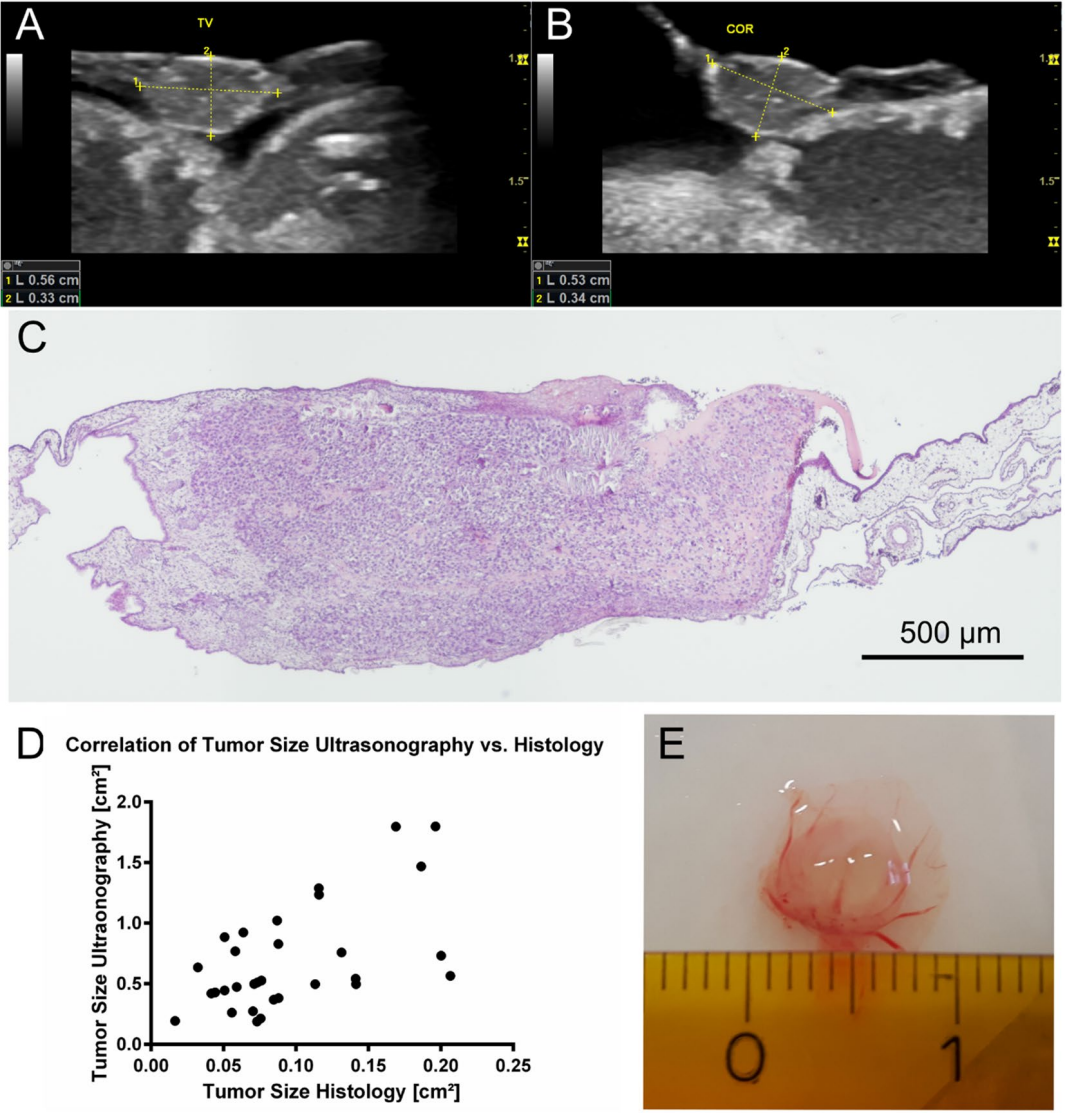
Tumor size in ultrasonography and histological slides: Ultrasonographic image of the inoculated tumor in ovo in longitudinal (**A**) coronar (**B**) plain. Image of tumor in HE staining in light microscopy (**C**). Correlation of tumor size determined in ultrasonography and histology (r = 0.49) (**D**). Photographic evaluation of tumor size after excision on day 14 (**E**).

In ultrasonographic analysis the median tumor volume on the CAM determined on day 14 was 0.075 cm^3^ (± 0.072 cm^3^). Accordingly, the calculated two-dimensional tumor size was 0.69 cm^2^ (± 0.4355 cm^2^). Measurement of tumor size in histology determined an average tumor size of 0.096 cm^2^ (± 0.052 cm^2^). For both methodological entities the measured tumor sizes correlated significantly (r = 0.49, P[two-tailed] = 0.0047) in the Spearman test (Fig. 3).

### Tumor vascularization

**Figure 4:**
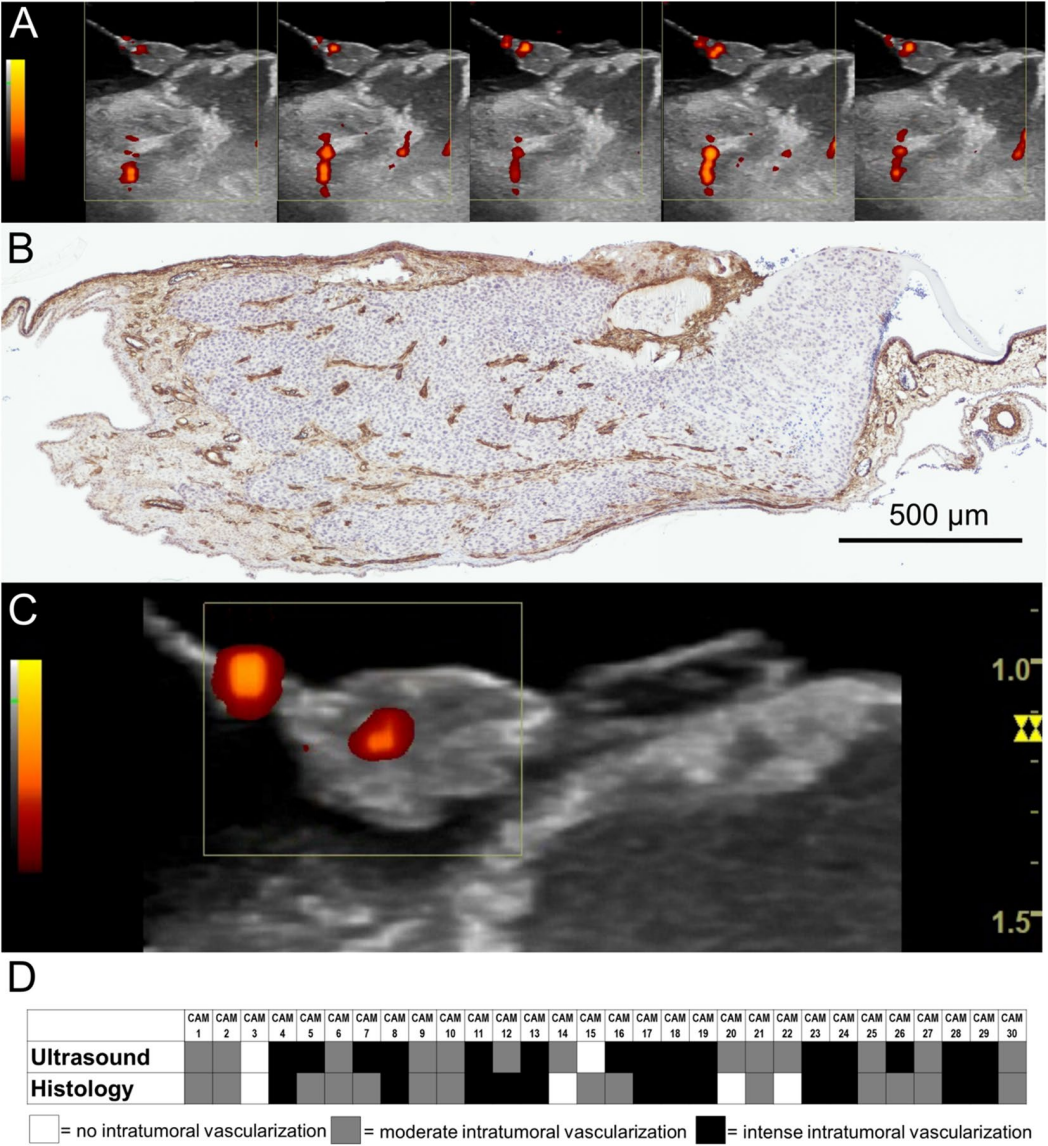
Tumor vascularization color-duplex- ultrasonography: Image sequence of the color-duplex- ultrasonography visualizing the intratumoral blood flow as well as the blood flow in adjacent anatomical structures *in ovo*. (corresponding video sequence attached. See Supplementary Information S1 (**A**). Histological section in ASMA staining with evidence of intratumoral vessel distribution (**B**). Intratumoral blood flow in color-duplex-ultrasonography. (Corresponding video sequence attached. See Supplementary Information S2) (**C**). Rating of presence and intensity of intratumoral vascularization, rated in a three step rating system (0 = no intratumoral vascularization, 1 = moderate intratumoral vascularization, 2 = intense intratumoral vascularization) (**D**).

Tumor vascularization could be visualized in 81.0% of eggs on day 14 using color-duplex-ultrasonography. 9.5% did not show any intratumoral vascularization, 28.6% were ranked as moderate intratumorally vascularized, and 61.9% showed an intense intratumoral vascularization. Analysis of histological slides showed vessel formation within the tumor tissue in 90.5% of all tumors analyzed. Comparable to the results obtained by ultrasonography 9.5% did not show any intratumoral vascularization. In 45% of cases the intratumoral vascularization was ranked as moderate and 47% of evaluated slides showed an intense intratumoral vascularization. Comparative analysis with the Spearman test regarding the intensity of vascularization within the tumor tissue correlated significantly (r = 0.65, P[two-tailed] < 0.0001) between ultrasonography and histological analysis (Fig. 4).

### Repetitive ultrasonography measurement

**Figure 5:**
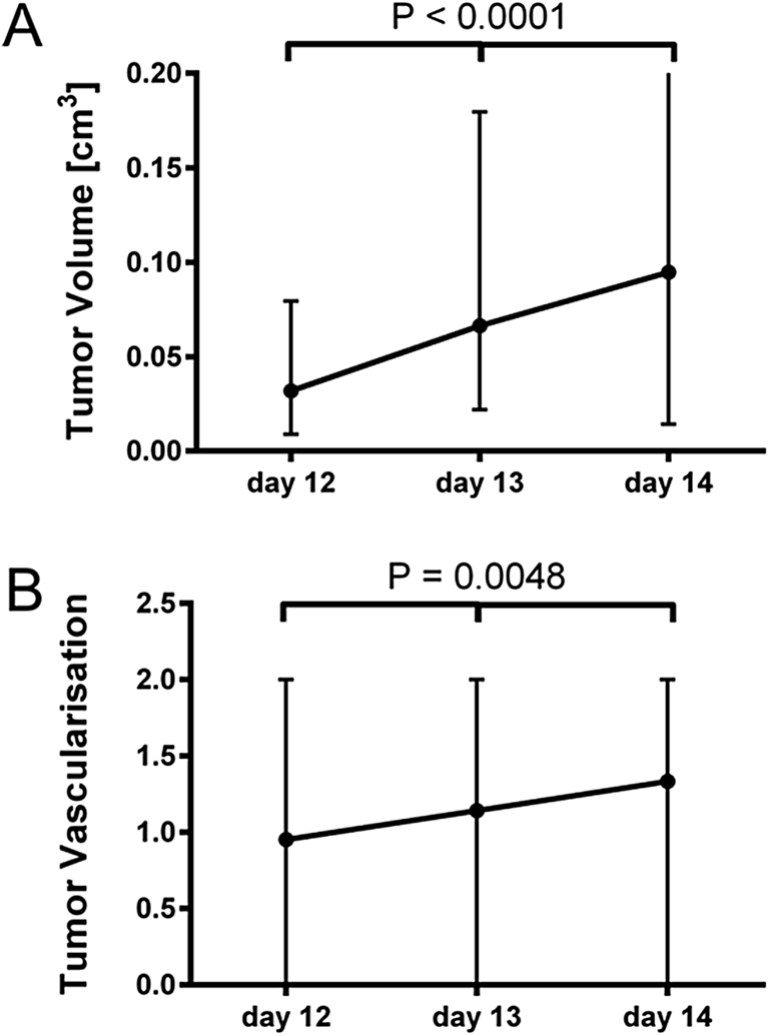
Longitudinal measurements of tumor size and vascularization in ultrasonography: Average tumor growth in repetitive measurements (shown as median + range). Differences between groups were calculated using the Friedman test (**A**) (n = 36). Repetitive evaluation of tumor vascularization in ultrasonography (shown as median + range). Differences between groups were calculated using the Friedman test (**B**) (n = 30)**.**

Repetitive ultrasonographic measurements on day 12, 13 and 14 of incubation revealed a significant increase of tumor volume within the timeframe of observation as shown in Fig. 5 (P < 0.0001). From day 12 to day 13 the tumors showed an average size increase of 129.5% while from day 13 to day 14 an average size increase of 45.5% was evaluated. Changes between the three days were significantly different as calculated with the Friedman test (p = 0.0048). Repetitive evaluation of tumor vascularization in ultrasonography showed an increasing perfusion of tumor tissue over the observed period of three days (Fig. 5).

### Survival analysis

**Figure 6:**
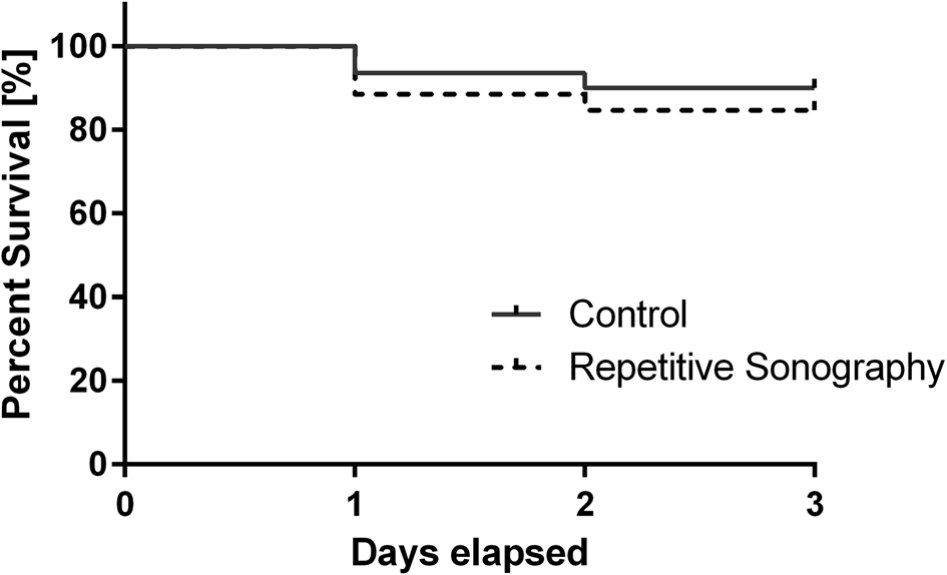
Dropout rates during experimentation: Kaplan Meier curve of dropout rates during the time period (day 12, 13, 14) of repetitive ultrasonography. The log-rank test as well as the Gehan–Breslow–Wilcoxon test were used to determine differences in dropout rates.

For repetitive ultrasonography, a log-rank test was performed to determine whether repetitive ultrasonography caused a significantly impaired survival when compared to untreated controls in the timeframe from day 12–14 of incubation. The differences in log-rank as well as the Gehan-Breslow-Wilcoxon test did not show significant differences for survival between the two groups (log-rank test [P = 0.42]; Gehan-Breslow-Wilcoxon test [P = 0.41]) as shown in Fig. 6.

### Cost calculation

Calculation of costs regarding different methodologies is difficult due to diverse prices for animals and housing in different institutions. Furthermore, additional costs like tumor cells, tools for preparation, housing or infrastructure may differ depending on the specific experimentation setup. Therefore, costs for cages, incubators, tumor cells, cell-medium or further equipment were not taken into consideration.

Furthermore, since we currently do not perform any experimentation with subcutaneously implanted tumors in rodents in our laboratory, inoculation rates from published data using liver cancer cell lines^38–43^ were utilized as comparative data. As shown in Table 1 median costs for a hen’s egg were approximately 2.1 € while a single nude mouse *(Crt:NU(NCr)-Foxn1nu/nu)* has an average cost of 53.8 € per animal. Of all the eggs incubated after shipment, 53% show successful tumor inoculation on day 11 after incubation. Although solid data regarding ingrowth of subcutaneously implanted tumors in scientific articles is scarce and has shown a large variety ranging from below 50%^42^ to 92% ^41^. The median inoculation rate was estimated around 77.5% (median value considering literature analysis).

**Table 1:** Kindly provide caption for the Table.

	CAM assay	Nude mouse^1^ (male, six weeks old)
Median costs/animal [€]	2.06^2^	53.84^3^
Successful tumor growth [%]	50	78^4^
Running costs / week [€]	0.02	2.94^5^
Calculated average cost/successful tumor (*in ovo/ *in vivo*)* [€]	4.13	69.47

^1^Resembled by the *Crt:NU(NCr)-Foxn1nu/nu* strain.

^2^Median costs/ egg (including transport costs) of the main distributer of Hens-Eggs in the specific region (Bio-Aufzucht LSL Rhein-Main GmbH, Dieburg, Germany).

^3^Median Costs for a male, six weeks old *Crt:NU(NCr)-Foxn1nu/nu* mouse according to the 3 available distributers for nude mice in the specific region (Janvier Labs, Paris, France; Charles River Wiga GmbH, Sulzfeld, Germany; Envigo RMS GmbH, Roßdorf, Germany).

^4^Gong et al.^39^, Huang et al. ^40^, Robertson et al.^41^, Xu et al.^42^ and Zhang et al.^43^.

^5^Resembled by the average running costs for SCID mice in our specific institution (Translational Animal Research Center, Gutenberg University Mainz).

Taking these numbers into consideration and adding the running costs like food and housing for two weeks into consideration, the average price for an egg bearing a tumor on the CAM is 4.1 € while a mouse with a subcutaneous tumor will cost approximately 69.5 € per animal (Table 1).

## Discussion

In recent years, the CAM-assay has been established for many scientific purposes. In tumor experimentation however, rodent experiments with subcutaneously implanted xenografts are still considered the “gold standard” regarding the evaluation of tumor treatment. In our working group the CAM-assay is frequently used for tumor experimentation. As shown in Fig. 2, 50.39% (± 11.46%) of the eggs incubated with tumors showed viability as well as sufficient ingrowth of the transplanted tumor on day 11. Tumor take rates reported in scientific literature show a large variety and range from around 45%^44^ to much higher inoculation rates of 70–80%^20, 32, 45^ depending on the specific tumor entity used for experimentation. Unfortunately, in many studies “true” dropout rates are not sufficiently reported and only positive ingroates or treatment effects are documented.

In our experience, differences in dropout rates are likely to be attributable to varying fertilization rates or possible blunt damages to the egg during transportation. During experimentation, main reasons for dropout include insufficient ingrowth, luxation or movement of the tumor on the CAM as well death of the chicken embryo. Furthermore, the eligibility for ultrasonographic imaging is heavily determined by the placement of the tumor on the CAM. Approximately 54.0% (± 29.47%) of the eggs bearing an inoculated tumor on the CAM meet this criterion and ultrasonographic imaging and measuring is possible in all three dimensions (Fig. 2).

Different working groups have published data describing the evaluation of tumor growth using the CAM-assay. In many publications however, tumor growth is only measured two-dimensionally or estimated using surrogate parameters like bioluminescence^32^. Furthermore end-point-analysis by means of size measuring^46^, cell count in flow cytometry of digested tumors, or weight measurements^47^ have been utilized for the estimation of tumor size.

Interestingly, accurate methodologies incorporating three-dimensional imaging have rarely been published. Kim et al. used MRI imaging for anatomical studies^48^ and Henning et al. used contrast agents to visualize the chick anatomy *in ovo* with computed tomography^35^. Recently, Huang et al. compared analysis with ultrafast ultrasound microvessel imaging (UMI) with power Doppler imaging^49^ *in ovo*. Since only a singular time point was analyzed in all studies mentioned above, repetitive longitudinal monitoring of tumor size and monitoring of tumor growth has not been previously described.

As shown in Fig. 5 we were able to repetitively analyze tumor size and vascularization using a commercial ultrasonographic scanner for human use. As ultrasound is commonly used in clinical diagnosis, many scientific institutions attached to hospitals have access to this imaging technique. Additionally, operating costs are rather low, the methodology is time-efficient and there is no need to apply potentially harmful contrast agents.

With regard to the assessment of the tumor size using ultrasonographic imaging compared to the histological sectional image, we expected great inaccuracies due to deviations in the sectional plane, artefacts and shrinkage during the preparation of histological slides. Nevertheless, tumor size in a two-dimensional sectional plane significantly correlated between ultrasonographic imaging and histological analysis (Fig. 3). The average plane of the tumors was much lower in histological analysis (13%) when compared to ultrasonographic measurements. Hence, we assume an intense shrinkage of the specimen during the fixation process. This is further supported by the fact that photographic documentation of the tumors showed diameters similar to ultrasonographic measurements far exceeding the measurements obtained from histological specimen (Fig. 3). The accuracy of the ultrasonographic measurements was further evaluated indicating an inaccuracy of approximately 1% compared to caliper measurements (Supplementary Information S2). A positive correlation was also determined between the three-dimensional tumor volume in ultrasonography and the tumor area determined in the histological slide (r = 0.48, P[two-tailed] = 0.0059) (Supplementary Information S3). By implication, we strongly believe that tumor size can be sufficiently monitored using ultrasonographic imaging.

Additionally, repetitive visualization of tumor vascularization further allows the monitoring of tumor perfusion (Fig. 4). This is immensely useful since therapeutical effects on intratumoral vessels (e.g. during radiotherapy or after application of anti-angiogenic drugs) can be longitudinally quantified^40, 50^.

Using repetitive ultrasonographic imaging, tumor angiogenesis, resembled by an increase in tumor vascularization also was visualized (Fig. 5). Similar to the findings reported by Huang et al.^49^ tumor vascularization also correlated with the findings obtained in histological analysis. Using a three step rating system for intratumoral vascularization, we were able to show a significant correlation (r = 0.65, P[two-tailed] = 0.0001) between findings obtained in ultrasonography and histological analysis (Fig. 4).

Even though increase in tumor-size of this magnitude was not expected (Fig. 5), other working groups have reported similar courses after penetration of the tumor by new blood vessels^51^. These findings are further supported by the increase of detectable vascularization in color-duplex-ultrasonography indicating a progredient supply of oxygen and nutrients within the tumor (Fig. 5).

In contrast to radiological imaging like CT, ultrasound represents a cost-efficient alternative without the effects of ionizing radiation. In addition, CT scans evaluating soft tissue regions, highly depend on the application of contrast agents. Contrary to CT, MRI does not require radiation or contrast agents. Yet, the increased time and cost factor as well as impaired imaging resolution due to movement of the embryo are obvious methodological limitations of compared to ultrasonography^48^.

3–5 ml of NaCl on the CAM are necessary for transduction of ultrasound waves to the CAM. As the fluid itself or the higher hydrostatic pressure might influence the experimental protocol, dropout rates were determined for all eggs undergoing repetitive ultrasonographic imaging as well as untreated eggs. As shown in Fig. 5 no significant differences in the dropout rate were detected during the respective timeframe.

Obviously, quality of imaging could be improved by using high resolution imaging like UMI^49^. However, ultra-high frequency imaging systems represent highly specialized and costly research equipment which is not available in most institutions. In contrast, commercial ultrasonographic scanners are pretty much omnipresent in clinical patient care.

The comparison of tumor size between ultrasonography and histological slides has obvious drawbacks like folding artefacts, shrinkage of tissue and deviations in the sectional plane. As tumors inoculated in the CAM often show extensive hematomas as well as intense ingrowth of the CAM’s stromal cells into the tumor tissue, increase of the macroscopic tumor may not be attributable to growth of tumor tissue itself but rather by a bidirectional infiltration of tumor tissue and CAM and accumulation of fluid by means of hematoma^27^. In rodents however, due to stromal cell invasion^52^ measurement of tumor size also bears similar chances of inaccuracy of measurements of subcutaneously implanted tumors. Although subcutaneously implanted tumors do rarely infiltrate the adjacent tissue e.g. muscle and stroma, measuring with calipers-based measurement bares the possibility of inaccuracy due to deviations in transtumoral measuring axis and inter-operator variability^53, 54^. Mouse models have a widespread availability, fast reproduction rate as well as relatively low housing costs compared to larger laboratory animals. Furthermore, the availability of multiple knock out and transgenic lines makes them the favorable animal model for most researchers. According to the German Ministry of Food and Agriculture, in 2017, 1.37 million mice were used for animal experimentation in Germany alone, making them by far the most frequently used vertebrate species in research today. Xenografts are usually implanted subcutaneously. In most tumor experiments three-dimensional size monitoring is realized using calipers or advanced imaging like CT, MRI, or bioluminescence detection^55^. Unfortunately, any evaluation of the tumor size in rodents results in stress or discomfort for the animal through repetitive handling, movement restriction, intravenous/intraperitoneal application or sedation. In contrast, in the CAM-assay neither the CAM nor the embryo are nociceptive. This not only makes *in ovo* experimentation ethically more justifiable but reduces the influence of factors like pain and stress on the experimental outcome. The short observational window may be considered as an obvious disadvantage of *in ovo* experimentation as the hatching of the chicken on day 21 will ultimately terminate the investigational timeframe. In contrast, rodent experiments allow much longer therapeutic and observational timeframes. Due to ethical concerns and the occurrence of nonspecific inflammatory reactions after 15 days of incubation^2^, we only evaluated tumor growth in the CAM-assay till day 14 after incubation. However, other working groups have published research evaluating tumors grown in ovo up to day 20 of incubation^56^. Additionally, tumors on the CAM grow much faster than equivalent tumors subcutaneously implanted into mice. Hence, vessel ingrowth^51^ as well as tumor metastasis were successfully determined using the CAM-assay^57^ allowing a sufficient monitoring of tumor development, regardless of the limited observational time frame. Aside from Hu et al. who observed equivalent tumor growth patterns and metastatic behavior for renal carcinoma cells in the CAM-assay and in mice^56^, further working groups were able to demonstrate parallels regarding the tumor size for different tumor entities in comparative experiments^46, 57^. In our experience, a major disadvantage of experimentation with a chicken model is the very limited commercial availability of chicken specific antibodies. With increasing popularity of this model however, this may change in the near future. Furthermore, detection of the intratumoral vessels is obviously limited by the sensitivity and resolution capacity of commercial ultrasonographic scanners. Rodent models like the dorsal skinfold chamber^58–60^ may allow an exposition of tumoral tissue similar to the CAM-assay, yet dropout rates and the severity of surgical intervention far exceed effects of subcutaneous tumor implantation^61^. Obviously, factors like inter-operator variability and a learning curve apply to ultrasonographic imaging as well. However, experienced examiners exist throughout multiple medical disciplines. Furthermore, the very simple methodology and visual identification of the tumor, due to its distinct shape on the CAM, allow a rapid acquisition of the skillset necessary for implementation of this technique. Despite its limitations, the CAM-assay is a versatile model offering a translational significance similar to equivalent rodent experiments for many scientific questions in the field of tumor research.

Chicken embryos are not considered independently living animals in most countries. Use of the CAM–assay is therefore not classified as an animal experiment*.* Still, obvious ethical concerns regarding the replacement of an animal experiment with another animal experiment remain and have to be addressed carefully. However, due to the lack of nociception in the CAM-assay as well as the embryo until day 14, this methodology is ethically preferable to homologous mouse experiments.

As exemplarily shown in Table 1 the costs for a sufficiently ingrown tumor suitable for experimentation are quite low, emphasizing the significantly increased cost efficiency of the CAM-assay compared to mice-experiments. Even though other institutions might be able to obtain mice for low prices or keep animals at moderate running costs, the CAM-assay has obvious economic benefits regardless of lower tumor inoculation rates. Furthermore, keeping chicken embryos does not require a governmental approval to keep and breed experimental animals. Therefore, this model can also be used in laboratories that do not have an animal experimental unit. Due to a high biological variation, animal experimentation is influenced by both genotype and environmental conditions resulting in an impaired reproducibility^62, 63^. Standardization of experimental setup may be one way to address this problem^64, 65^. However, effects of biological variability might heavily influence experimental outcome especially if small sample sizes are utilized. Taking these factors into consideration, the CAM-assay may offer some key advantages. Biological variability due to behavioral aspects and influences like social interaction, stress and anxiety are much less present in hatching chicken eggs due to the limited sensorineural input. Secondly, the rather simple methodology facilitates a good standardization and reproducibility of experimental conditions. This leads to a reduced inter-operator bias and a lower time expenditure compared to rodent–xenograft-models. Especially the lower time expenditure may facilitate experimental efficiency which directly translates to an increased quantitative output. All these advantages might contribute to an increased reproducibility of experimentation especially in comparison to equivalent rodent-xenograft-models.

## Conclusion

In conclusion, repetitive ultrasonography is suited for sufficient quantification of tumor size and monitoring of intratumoral vascularization without increased dropout rates. For the first time, tumor growth and tumor angiogenesis have been successfully visualized *in ovo*. Using the CAM-assay for tumor research has obvious advantages like time- and cost-efficiency as well as widespread availability resulting in a high quantitative output and an increased reproducibility. Therefore, ultrasonographic imaging further diversifies the applicability of the CAM–assay as an alternative to homologue rodent models in the field of tumor experimentation.

## Data availability

The datasets generated during and/or analyzed during the current study are available from the corresponding author on reasonable request.

## Supplementary information

Supplementary Information 1.

Supplementary Video 1.

Supplementary Video 2.
