# Supplementary material for: Monitoring of tumor growth and vascularization with repetitive ultrasonography in the chicken chorioallantoic-membrane-assay
Source: Sci Rep. 2020 Oct 29;10:18585. doi: 10.1038/s41598-020-75660-y (PMC7596505; doi:10.1038/s41598-020-75660-y)
Supplement: Supplementary file 1 — Supplementary Information 1. [file 41598_2020_75660_MOESM1_ESM.pdf]

Supplementary Files:

Monitoring of Tumor Growth and Vascularization with Repetitive Ultrasonography in the Chicken Chorioallantoic-Membrane-Assay.

Jonas Eckrich, Philipp Kugler, Christoph Raphael Buhr, Benjamin Philipp Ernst, Simone Mendler, Jan Baumgart, Juergen Brieger, Nadine Wiesmann

Supplementary Video S1) Intratumoral Blood Flow (1)

Supplementary Video S1: Video sequence of the color-duplex-ultrasonography visualizing the intratumoral blood flow as well as the blood flow in adjacent anatomical structures in ovo.

Supplementary Video S2) Intratumoral Blood Flow (2)

Supplementary Video S2: Video sequence visualizing the intratumoral blood flow in color-duplex-ultrasonography

Supplemantary Note S3) Accuracy of Ultrasound Measurements

Objective: Accuracy of ultrasonographic measurements was determined by measuring ten different well standardized objects (gummi bears) with both ultrasound as well as caliper measurements.

Methods: Gummi bears (Haribo-Holding GmbH & Co. KG, Bonn, Germany) due to there standardized size and homogeny were immersed in a graduated beaker filled with water to a level of 1.5 cm and individually measured using ultrasonographic imaging. The maximum axis in both longitudinal (l) as well as transversal (t) and coronar (c) diameters were determined. After sonographic measurements, the gummi bear was taken out of the water and measured again using a commercial caliper (Brüder Mannesmann, Remscheid, Germany) (Figure S3).

Results: In ultrasound measurements gummi bears had median maximum diameters of 1.99 cm (l) x 1.09 cm (t) x 1.32 cm (c) compared to median diameters of 2.05 cm (l) x 1.10 cm (t) x 1.35 cm (c) in caliper measurements (Table S3). Median difference between the two measuring techniques regarding length of all three axes was approximately 1%.

Conclusion: In an exemplary setting measurements with ultrasound are very accurate showing only a minimal divergence (~1%) compared to caliper measurements

Figure S3) Methodology of Ultrasonographic and Caliper Measurement

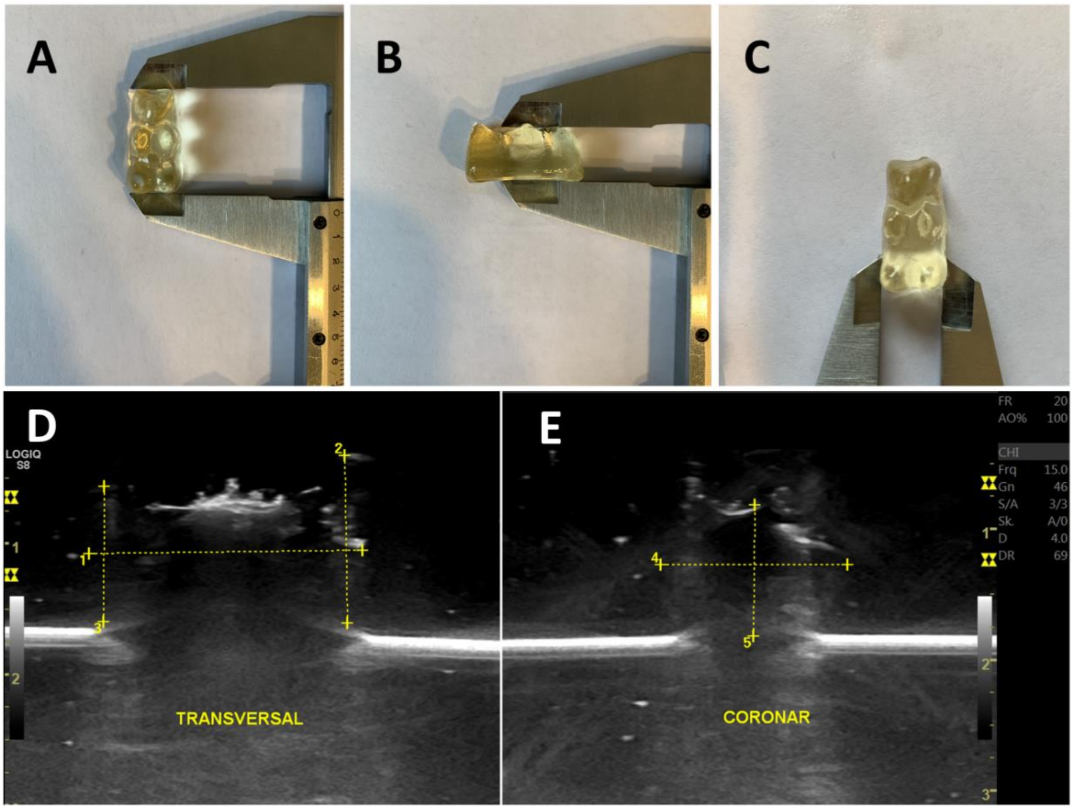

Figure S3: Caliper measurements of the gummi bear after ultrasonography **(A-C)**. Ultrasonographic visualization and measurement of the gummi bear immersed in water **(D-E)**.

Table S3) Comparative Analysis of Ultrasonographic and Caliper Measurement:

**N=10**	**Ultrasonography**			**Caliper measurement**			**Median difference [%]**
	longitudinal	transversal	coronar	longitudinal	transversal	coronar	
**Median length [cm]**	**2.00** (1.96-2.10)	**1.09** (0.98-1.29)	**1.32** (1.10-1.43)	**2.05** (2.00-2.10)	**1.10** (1.00-1.30)	**1.35** (1.00-1.45)	**1.00** (-2.5-5.2)

Table S3: Comparative analysis of measurements of the immersed gummi bears with both ultrasonography as well as caliper measurement. Data shown as median (range).

Figure S4) Correlation of Tumor Volume Sonography vs. Histology

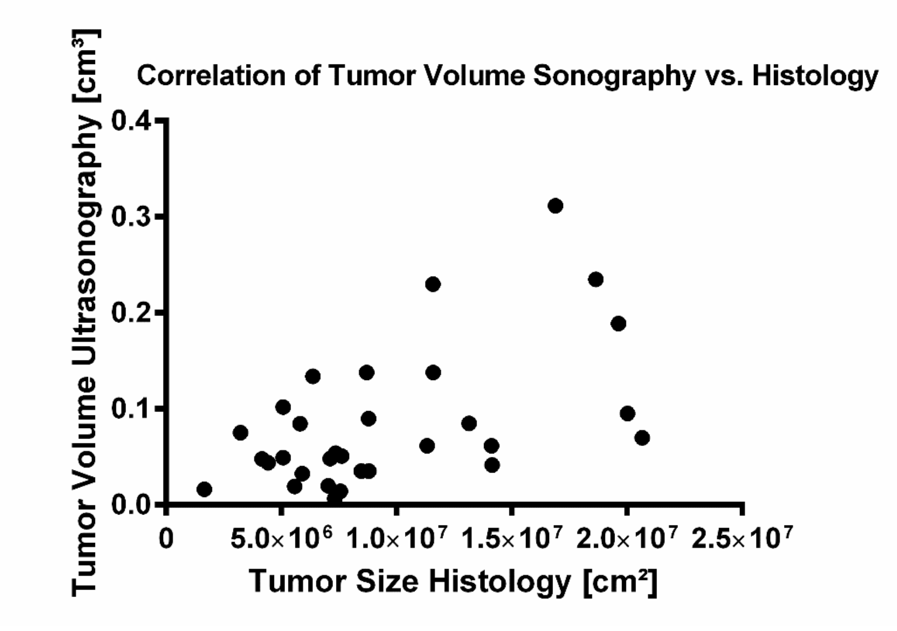

Figure S4: Correlation of tumor volume determined in ultrasonography and histology (r=0.48). Correlation was quantified using the Spearman rank correlation.
